# A parent's guide to promoting positive body image in their young children: development of a prevention resource

**DOI:** 10.1186/2050-2974-1-S1-O40

**Published:** 2013-11-14

**Authors:** Stephanie R Damiano, Chelsea Cornell, Laura Hart, Fiona Sutherland, Susan J Paxton

**Affiliations:** 1 School of Psychological Science, La Trobe University, Australia

## 

Body dissatisfaction (BD) and eating disorders (EDs) commonly emerge during adolescence, but foundations for these problems are laid during childhood. Also, some research has demonstrated that BD is increasingly prevalent in younger children. Parents play an important role in fostering positive environments for their children; however, parents need information and skills to facilitate this. This presentation describes the development of a resource to support parents in this task.

First, a literature review identified parental risk factors for child BD and EDs. Second, focus groups with 43 parents of children aged 1 to 6 years and interviews with 11 early childhood professionals highlighted that parents require more information about body image (BI) and the roles they can play in promoting children's positive BI. A concise and evidence-based resource with realistic guidelines in multiple formats was favoured.

Accordingly, a resource has been developed in booklet and website formats, providing parents with information about what influences a child's BI and practical tips to help their child develop positive BI. There are also several parent and child activities, all of which will be described. The resource is being evaluated using a randomised controlled trial with parents of children aged between 2 and 6 years.

This abstract was presented in the **Prevention** stream of the 2013 ANZAED Conference.

